# The views of young children in the UK about obesity, body size, shape and weight: a systematic review

**DOI:** 10.1186/1471-2458-11-188

**Published:** 2011-03-25

**Authors:** Rebecca Rees, Kathryn Oliver, Jenny Woodman, James Thomas

**Affiliations:** 1 EPPI-Centre, Social Science Research Unit, Institute of Education, 18 Woburn Square, London, WC1H 0NR, UK; 2 Centre for Occupational and Environmental Health, University of Manchester, Oxford Road, Manchester M13 9PL, UK; 3 MRC Centre of Epidemiology for Child Health, UCL-Institute of Child Health, 30 Guilford Street, London WC1N 1EH, UK

## Abstract

**Background:**

There are high levels of concern about childhood obesity, with obese children being at higher risk of poorer health both in the short and longer terms. Children's attitudes to, and beliefs about, their bodies have also raised concern. Children themselves have a stake in this debate; their perspectives on this issue can inform the ways in which interventions aim to work.

This systematic review of qualitative and quantitative research aimed to explore the views of UK children about the meanings of obesity and body size, shape or weight and their own experiences of these issues.

**Methods:**

We conducted sensitive searches of electronic databases and specialist websites, and contacted experts. We included studies published from the start of 1997 which reported the perspectives of UK children aged 4-11 about obesity or body size, shape or weight, and which described key aspects of their methods. Included studies were coded and quality-assessed by two reviewers independently.

Findings were synthesised in two analyses: i) an interpretive synthesis of findings from open-ended questions; and ii) an aggregative synthesis of findings from closed questions. We juxtaposed the findings from the two syntheses. The effect of excluding the lowest quality studies was explored. We also consulted young people to explore the credibility of a subset of findings.

**Results:**

We included 28 studies. Instead of a focus on health, children emphasised the social impact of body size, describing experiences and awareness of abuse and isolation for children with a greater weight. Body size was seen as under the individual's control and children attributed negative characteristics to overweight people. Children actively assessed their own size; many wished their bodies were different and some were anxious about their shape.

Reviewers judged that children's engagement and participation in discussion had only rarely been supported in the included studies, and few study findings had depth or breadth.

**Conclusions:**

Initiatives need to consider the social aspects of obesity, in particular unhelpful beliefs, attitudes and discriminatory behaviours around body size. Researchers and policy-makers should involve children actively and seek their views on appropriate forms of support around this issue.

## Background

There are high levels of concern about obesity in Westernised societies, and about obesity in children in particular [1]. The National Child Measurement Programme (NCMP) was implemented in the UK in order to monitor changes in average body size amongst children who are starting or about to leave primary education. In 2008/9 this classified almost one in ten (9.6%) children aged 4-5 as obese and, for 10-11 year olds, almost one in five (18.3% [2]. Children are likely to experience immediate physical and psychosocial problems as a result of being obese and are at a higher risk of obesity as they grow older [3]. Children's attitudes to and beliefs about their bodies, which can include high levels of body dissatisfaction, have also raised concern [4,5].

The right of children to participate in decision-making that relates to them is enshrined in the United Nations Convention on the Rights of the Child [6]. Children, like other members of society, have a stake in debates about body size, and their ideas about health and other aspects of their lives are increasingly seen as valid contributions to the development of social policy [7,8]. As well as leading to more effective policies and better services, research and policy-making that involves children actively in debate and decision-making can provide them with experiences of collective work and create a culture of shared responsibility and mutual trust [9]. Recent initiatives that act on this include one that has involved young people as advisers to a nationwide public health research programme [10].

Systematic reviews of intervention research indicate that there is a dearth of evidence from well-conducted studies to help us decide what can be done to prevent or deal with obesity [11,12]. Although children's own perspectives on obesity and body size, shape and weight can inform the ways in which interventions aim to bring about positive outcomes, there has been no previous attempt to bring studies of children's perspectives together. This systematic review aims to address this gap and to examine recent research findings where children aged from four to eleven provide views about their own body sizes or about the body sizes of others. The review sought studies conducted recently in the UK with the aim of informing policy development and the commissioning of further research in the UK. An additional review was conducted of studies of young people aged 12-18, as it was considered that perspectives might differ considerably between these two groups (at the time of writing, the report of this separate review is completed but under peer review).

This review addressed the following questions:

• What are children's views about the meanings of obesity or body size, shape or weight (including their perceptions of their own body size), and what experiences do they describe relating to these issues?

• What are children's views about influences on body size?

• What are children's views about changes that may help them to achieve or maintain a healthy weight?

## Methods

### Inclusion criteria

To be included in the review, studies had to report views about obesity, body size, shape or weight, sought from children in the UK aged four to eleven. Reports needed as a minimum to have described one of two key aspects of a study's methods (either data collection or analysis). They needed to have been published in English since the start of 1997 (to cover this recent period of heightened public health, research and media interest in the topic of obesity). Studies where all children had multiple, complex health needs were excluded.

We defined *views *as attitudes, opinions, beliefs, feelings, understandings or experiences, and excluded studies that measured only health or weight status, behaviour or factual knowledge. Views could be reported as a point on a scale, as agreement with a statement, as an answer to a closed question or as answers to, and discussion around, open-ended questions.

The inclusion criteria were initially piloted by four reviewers and a sample of early screening decisions was double-checked by the first-named author at the start of the screening process. Screening was then done individually by the four reviewers.

### Search strategy

During June and July 2009, we searched 18 electronic databases from the fields of health, public health, education, social science and social care, taking care to include sources rich in UK-based journal and report literature. To supplement this, we searched three key journals and sixteen websites by hand, scanned reference lists, looked for papers that had cited key studies, and contacted key informants for relevant research. Additional file 1 (online) details the search strategy in full. We managed review data using our specialised online review software EPPI-Reviewer [13].

### Describing and appraising studies

We described the final set of included studies using a standardised classification system developed for public health and health promotion research [14], and another set of questions which built upon frameworks used in previous reviews of the views of children and young people [15].

We appraised the quality of included studies using criteria modified from a set developed for examining the findings of evaluations of intervention processes (Table 1) [16,17]. As a final step in quality assessment, the studies were each allocated a 'weight of evidence' in two dimensions. First, we assigned a weight (low, medium or high) to rate the reliability or trustworthiness of the findings (the extent to which the methods employed were rigorous and minimized bias and error). This weighting focused on the criteria numbered 1 to 4. Secondly, we assigned a weight to rate the usefulness of the findings within our review (based on the richness and complexity of the description and analysis of children's views, and whether or not the data threw light on children's own perspectives of body size). This weighting focused on the criteria numbered 5 and 6. For all of the tasks, two reviewers worked independently on each study, then compared their descriptions and judgements, and came to a consensus.

**Table 1:** Criteria used to appraise study quality

**Criterion ***(with guidance for reviewers)*
1 Were steps taken to increase rigour in the sampling?
*Consider whether:*
*the sampling strategy was appropriate to the questions posed in the study (e.g. was the strategy well reasoned and justified?);
*attempts were made to obtain a diverse sample of the population in question (think about who might have been excluded; who may have had a different perspective to offer);
*characteristics of the sample critical to the understanding of the study context and findings were presented (i.e. do we know who the participants were in terms of, for example, basic socio-demographics, characteristics relevant to the context of the study, etc.).

2 Were steps taken to increase rigour in the data collected?
*Consider whether:*
*data collection tools were piloted/(and if quantitative) validated;
*(if qualitative) data collection was comprehensive, flexible and/or sensitive enough to provide a complete and/or vivid and rich description of people's perspectives and experiences (e.g. did the researchers spend sufficient time at the site/with participants? Did they keep 'following up'? Was more than one method of data collection used?);
* steps were taken to ensure that all participants were able and willing to contribute (e.g. processes for consent, language barriers, power relations between adults and children/young people).

3 Were steps taken to increase rigour in the analysis of the data?
*Consider whether:*
* data analysis methods were systematic (e.g. was a method described/can a method be discerned?);
*diversity in perspective was explored;
* (if qualitative) the analysis was balanced in the extent to which it was guided by preconceptions or by the data);
*the analysis sought to rule out alternative explanations for findings (in qualitative research this could be done by, for example, searching for negative cases/exceptions, feeding back preliminary results to participants, asking a colleague to review the data, or reflexivity; in quantitative research this may be done by, for example, significance testing).

4 Were the findings of the study grounded in/supported by the data?
*Consider whether:*
*enough data are presented to show how the authors arrived at their findings;
*the data presented fit the interpretation/support claims about patterns in data;
*the data presented illuminate/illustrate the findings;
*(for qualitative studies) quotes are numbered or otherwise identified and the reader can see that they don't just come from one or two people.

5 Please rate the findings of the study in terms of their breadth and depth.
*Consider whether:*
*(NB: it may be helpful to consider 'breadth' as the extent of description and 'depth' as the extent to which data has been transformed/analysed);*
*a range of issues are covered;
* the perspectives of participants are fully explored in terms of breadth (contrast of two or more perspectives) and depth (insight into a single perspective);
*richness and complexity has been portrayed (e.g. variation explained, meanings illuminated);
*there has been theoretical/conceptual development.

6 To what extent does the study privilege the perspectives and experiences of children?
*Consider:*
* whether there was a balance between open-ended and fixed response options;
*whether children were involved in designing the research;
* whether there was a balance between the use of an a priori coding framework and induction in the analysis;
*the position of the researchers (did they consider it important to listen to the perspectives of children?);
* whether steps were taken to assure confidentiality and put young people at ease.

7 Overall, what weight would you assign to this study in terms of the reliability/trustworthiness of its findings?
*Guidance:*
*Think (mainly) about the answers you have given to questions 1 to 4 above*.

8 What weight would you assign to this study in terms of the usefulness of its findings for this review?
*Guidance:*
*Think (mainly) about the answers you have given to questions 5 and 6 above and consider:*
*the match between the study aims and findings and the aims and purpose of the synthesis;
*its conceptual depth/explanatory power.

### Analysis

We synthesized findings in two separate analyses. One aimed to develop conceptual themes. This used the findings of studies where children had been enabled, by open-ended questioning, to describe their views in their own words. We labelled this synthesis 'interpretive' to reflect both the methods employed in the primary studies and those used to synthesise them (see below). The second synthesis used findings where children selected from responses already set by researchers. These researchers then explored variation in responses, for example in terms of differences in age, gender or Body Mass Index (BMI). We labelled this second synthesis 'aggregative', to capture the way that it, and the studies within it, primarily aimed to summarise data.

Two reviewers worked on the syntheses. In the interpretive synthesis, we used *thematic synthesis *to examine each line of each study's findings and create codes that described their meaning and content [18]. We then looked for similarities and differences between codes, grouped them into a hierarchical tree structure and wrote a narrative to illustrate each theme. For the aggregative synthesis, we grouped analyses into categories according to the type of view that had been analysed and then wrote a descriptive account of the reported findings [19]. We then looked at each finding from the two syntheses in turn to see if it related to a theme or question in the other synthesis. This brought together findings about perceptions and experiences of body size rooted in children's perspectives with findings about the circumstances in which children might hold these and other kinds of views about body size.

We drew up a table for each synthesis to count the number of themes to which each study contributed. This was then examined to see how much influence the lowest quality studies had on the syntheses.

### User involvement

The study was designed with the assistance of an expert steering group that included a representative from the review's funder. The group provided feedback on the review protocol.

We held a consultation with young people that explored the credibility of part of the review's findings. The consultation was held in two workshops organised by PEAR, a group established by the National Children's Bureau to enable young people's views and opinions to influence public health research [20]. After a brief discussion of the review's aims, participants in each workshop discussed the themes developed in the interpretive synthesis. They were asked whether they seemed believable and whether they thought any important themes were missing [19].

## Results

This section describes the focus and quality of recent UK-based research that has asked children for their views on body size. It then presents the substantive findings, and ends with findings from the consultation and the sensitivity analysis.

### The state of the literature: in what ways have children in the UK been asked for their views about body size?

A total of 11,128 citations were identified and screened for relevance and 28 studies were found that could be incorporated into the review's syntheses (Additional file 2 - online - details the process of excluding studies).

These studies varied considerably in terms of their stated aims and collection of data (see Additional file 3 - online - for a description of each study). Of the 15 studies in the interpretive synthesis, only seven focused directly on body size, shape or weight, or the act of measuring BMI [21-27]. Five studies explored a variety of perceptions about either physical activity [28,29] or children's eating [30-32]. Three were focused more broadly on mental health, or on health as a whole [33-35]. The 13 studies in the aggregative synthesis [36-48] were in the main focused on attitudes towards body size, and explored the relationships between children's views (for example, perceptions of their own size, body shape ideals, satisfaction and stereotyping responses) and demographic variables such as gender and age.

We found no studies that asked children directly what they thought should be done to help them to reach or maintain a healthy size. The rest of this paper reports findings about the meanings of body size for children (including their perceptions about their own body size) and their reported experiences in this area. Findings about children's views about influences on body size are reported in full elsewhere [19].

Nearly all of the children in the 28 studies were recruited through their schools and were described as having a normal range of body sizes, or were not characterized at all in these terms. Only three studies aimed to study children with very high body sizes [22,24,27]. Children's ethnicity and socio-economic status were also frequently not stated by the study authors. Only eight studies included children aged under seven [25,28,29,33,35-38].

In terms of quality, very few studies were judged to have highly reliable findings (Additional files 4, 5 and 6 - online). For example, many studies providing data for the interpretive synthesis reported their data analysis methods only very briefly, or not at all. Few in the aggregative synthesis reported methods for increasing sampling rigour. Furthermore, many studies were judged to have taken only minimal steps to privilege children's perspectives; steps which were necessary to provide the most useful data for this review. Only a few, for example, appeared to have used questioning techniques that encouraged children to develop their own ideas.

Four studies in the interpretive synthesis [25,27,28,34] and two studies in the aggregative synthesis [38,48] were judged to be low in terms of both reliability and usefulness for those syntheses. An analysis of the potential influence of excluding the findings of these six studies from the synthesis can be found at the end of the results section.

### Substantive findings: what does body size mean to children and what are their perceptions and experiences of their own and others' body sizes?

The children's perceptions and experiences of their own and others' body sizes were grouped under four main themes: i) how body size is or is not a matter of importance in the world; ii) desirable and acceptable bodies; iii) embodied experiences (children's experiences of and feelings about their own body sizes); and iv) gender. A further 15 sub-themes arose from these findings (see Table 2) and the remainder of this section illustrates these in turn. The complete set of themes produced in the syntheses can be found in the review's technical report [19].

**Table 2:** The themes used to group children's perceptions and experiences of their own and others' body sizes

Major themes	Sub-themes
Body size matters	Body size might not always seem relevant
	Being overweight is seen as a social problem
	Body sizes are judged
	Discrimination is normal
	Children are aware that body size is a public issue

Desirable and acceptable bodies	Desirable bodies are not overweight
	A large body size means you are...
	Children apportion blame and responsibility for fat

Embodied experiences	Children actively assess their own size
	Discomfort and feelings of pressure accompany a focus on body size
	Children express dissatisfaction with their bodies
	The consequences of body size are experienced as social in nature
	Very overweight children are made to feel 'different and terrible'

Gender	Satisfaction with body size differs between the sexes
	Body size stereotypes vary with gender

### Body size matters

#### Body size might not always seem relevant

The salience of body size for children varied. Some did not mention body size at all when asked about issues that were important for them, or for their health or well-being [22,34,35]. It was thought that size might be a problem later in life, rather than in the present [21,32,35]. As one child put it,

'as a teenager you get fat and have other problems and that's when you need help most.'[[35]; p35]

Body size was, however, clearly highly central to many overweight children's lives. When encouraged to talk about any aspect of their lives, several very overweight children in one study introduced body size as the very first thing they wanted to talk about [22].

#### Being overweight is seen as a social problem

The main aspect of body size considered important by children, regardless of their own size, appeared to be how being large can affect popularity and fitting in. Body size was seen by children to affect both the way they interact with each other and how included they feel. Children thought that overweight children might not have people to play with, or be lonely [34], might be less popular than thin children [21,34], might only have fat friends [30], might need to choose a boyfriend or girlfriend the same size as themselves [21], or might even need to get slim in order to make friends [231, [22]].

#### Body sizes are judged

Some children referred to the idea that appearance should be discounted in favour of other characteristics [21,22,34], for example,

'if you're a good person on the inside, then it doesn't really matter how you look on the outside' [[34], p14].

But this was challenged by other children's accounts [21,22]. As two boys put it,

'It's not a very good image if you are going round with a fat person.' (Boy cutting in:) 'nowadays it's all on your looks' [[21], p10].

Another said,

'they say that now, but in real life they'll make fun of you if you're different' [[21], p210].

#### Discrimination is normal

Children thought that it was usual for overweight children to be treated differently because of their body size [22,23,31]. Summing up how such children could expect to be singled out, one child said,

'They'll be miserable for the rest of their lives because they'll get picked on' [[21], p209].

In one study, boys were said to regard teasing as a legitimate response if being overweight was someone's own fault [23]. Overweight children also reported believing that teasing would cease if they lost weight [27].

Boys described feeling conflicted about taking part in size-related ridicule, with one saying,

'Your mates pick on them and you join in, but you don't want to inside' [[21], p109].

In two studies, even those boys who had been teased or bullied themselves seemed accepting of it [21,24]. As one clinically obese boy said,

'You hear people calling them fat but that's just normal isn't it?'[[24], p921].

#### Children are aware that body size is a public issue

Children were aware of public interest in body size, and about common media representations. They described fictional and reality television programmes as influencing their awareness, sometimes providing information and sometimes contradicting reality [21,31,34]. One girl said,

'They make clothes for stick-thin people and in magazines everybody's thin and you don't get fat people in them' [[21], p210].

### Desirable and acceptable bodies

#### Desirable bodies are not overweight

Being overweight was almost always described as undesirable. A few positive comments were made by boys who noted that fat might help keep you warm [21,22] and that it was preferable to [21], or could help prevent [22] starvation.

Overall, children talked mainly in negative terms when they evaluated different sizes. Girls talked solely in favourable terms about having a 'slim' body [26,34] or 'a good figure' [31], but did not discuss this in any depth. Only one study encouraged boys to describe their aspirations for their bodies. In contrast to girls' emphasis on body fat, these boys expressed their ideal body as one that 'looked fit' [[23], p224].

Views about being underweight were more mixed. Children in several studies described how girls of their age might want to be 'thin' or 'skinny'[21,22,30]. Both boys and girls, however, also linked being 'too thin' unfavourably with anorexia [[31], p548] and girls linked it with being 'quite ill' [[21], p210] and 'obsessed'[[30], p21]. In one study, children aspired to the 'lovely wee skinny little bodies' of models and some media celebrities, while also judging some to be 'too thin' [[31], p549].

Several studies explored variations in children's preferred and aspired-to shapes. Differences between boys and girls are discussed in 'Gender', below. In one study, children's preferences had been contrasted with the measured averages for children's BMI [39]. Many of these children aspired to thin or very thin bodies, and over three-quarters wanted bodies the same or smaller than the 25% of children at the 'leanest' end of the scale. The boys' and girls' preferences in this study were similar.

#### A large body size means you are...

Children provided consistently negative evaluations of overweight people [26,36,38,40,41]. This was seen in children across a range of ages, and in some as young as five [36,38]. They attributed a wide variety of negative behavioural, personal and social characteristics to generalised representations of overweight children and adults, including: eating food and drinks with a high fat and/or sugar content [26,30,33]; not eating healthy food [33]; eating inedible or unrealistic food items [26]; laziness and watching too much television [30,33]; poor table manners [26]; not washing, not exercising [33]; 'trouble-making'[30]; and having no hobbies [30].

While children did provide researchers with generalisations about overweight people, only one study reported children's reflections on these ideas. In the following excerpt, two children, supported by a researcher, discussed an imaginary 'unhealthy child'. When encouraged to express themselves more fully, they indicated their understanding of the difficulties that overweight children might experience,

'She's fat, and ... really smelly' 'and she has bad breath' 'and she's always stroppy and stressed' 'so she doesn't have many friends' ... 'Well it's quite likely that a person who's fat would be selfish. I think. Do you think?'

(Adult directs back to other child - 'what do you think?')

'I think maybe [they would feel] stressed. So it would be hard to think about other kids.' 'Yeah it would be hard to run.' 'It would be hard to do anything.' 'You wouldn't feel good' [[33], p11].

#### Children apportion blame and responsibility for fat

Children talked judgementally about overweight children and adults. In one study, boys linked becoming overweight with a weakness of will and saw it as something that people had control over [23]. In another, children implied that being overweight could be a just punishment for this lack of control. One said, for example,

'[They] deserve to be fat if they eat sweets all the time' [[21], p209].

Children were viewed as more accountable if they had become overweight through self-indulgence or by eating the wrong food. They were less accountable if their size was linked with genetics, or was in some way 'natural'. For example, one child said,

'If it's weight they've put on, they should do something; if they're genetically like that, then they shouldn't' [[21], p209].

### Embodied experiences

#### Children actively assess their own size

Children described how they compared their bodies over time and with other children's bodies. Some studies explored the extent to which children were able to estimate their own body size, using pictorial scales that showed actual body sizes [26,39,41-43]. These found that children of all sizes generally produced relatively accurate estimations of their own body shapes.

Girls in one study commented that boys, when they appraise the degree to which someone is 'fat', sometimes start with a person's overall size and confuse muscularity and body fat [21]. They also said that if a boy was fat, they would describe themselves as 'muscly'. To illustrate, one boy is reported as saying,

'You could be fat and healthy like rugby players who have a lot of exercise' [[21], p211].

In two studies, very overweight children commented critically on others' assessment of their size [22,24]. In both cases, the children were disagreeing with their parents' evaluations of their bodies. One child, for example, said,

'My mum tells me that I'm not overweight, but I know I am' [[22], p235].

#### Discomfort and feelings of pressure accompany a focus on body size

Children described negative emotions around body size, including a generalized anxiety and pressure, regardless of their weight. In two studies, both boys and girls who probably had a healthy weight, expressed anxiety when asked about being measured [25,53]. The anxiety seemed to be about being labelled as an unacceptable size and disclosure of this information to peers. As one boy put it,

'I'd get scared and worried if the rest of the class were there in case you are fatter than you think you are' [[25], p5].

Children described their perceptions of pressures on girls in particular [21,30,31,34], saying, for example,

'When you think of boys you think of sweets, you think of chocolate ...Yeah, they think they are already strong so they don't have to go on any diets'[[30], p25].

Girls themselves sometimes expressed anxiety about their own bodies. They talked about feeling self-conscious on occasions when they had to expose more of their bodies - particularly when swimming, or feeling uncomfortable when swimming with a friend who was more developed physically [34]

Children may also experience a more diffuse sense of unease about body size. In two studies, researchers noted that children reacted unexpectedly to the topic of obesity [33,34]. In one, researchers found girls reluctant to talk about body size and appearance at all [34].

#### Children express dissatisfaction with their bodies

In many studies, children across a range of body sizes reported low levels of satisfaction with their bodies. The satisfaction of girls whose actual BMI classified them as overweight was consistently lower than that of other girls [22,26,43,44]. In contrast, two studies found that boys' satisfaction with their body shape sometimes increased when their actual body size rose above levels recognised to be healthy [22,26]. This difference is described further below (see 'Gender').

Only one study asked children what they did like about the shape and size of their bodies [26]. Just over half of these children, who were mainly neither overweight nor underweight, answered 'nothing'. In this study, 'tummies' were the body part liked the most. In contrast, the stomach was viewed by children who were overweight, in a separate study, as the only part of the body that was a problem [22].

Children's explanations for their dissatisfaction were not recorded, apart from in the case of one very overweight boy. Again, this child emphasised a desire to fit in, saying,

'I don't look very nice. When I'm dressed up I look all right ... slimmer ... I feel different from the others'[[22], p225].

However, body size could not always predict whether children would express dissatisfaction or satisfaction with their bodies. One girl, described as only moderately overweight, was moved to tears when she discussed how much she wanted to become thinner and so be accepted by a friendship group that she wished to join. But being very overweight did not mean that weight was necessarily described in negative terms. One very overweight girl, when asked if she would like to change anything about herself, said she was 'not really bothered'. This was corroborated by a friend, who said '[she] likes the way she is ... She don't mind' [[22], p232].

#### The consequences of body size are experienced as social in nature

Very overweight children had felt the negative social consequences of overweight described by their healthy weight peers. In one study, a third of overweight children described feeling less socially accepted than their peers. Fewer than one in ten children with a more optimal weight reported feeling this way [22]. One very overweight girl described how weight 'gets in the way' of her making friends [[22], p228] and one very overweight boy reported feeling disliked because of his weight. Others reported being abandoned by friends. Nearly three-quarters of obese children in another study felt that they would have more friends if they lost weight [27].

Overweight children reported that size-related abuse had led to negative changes in their behaviour. Some boys described how bullying led to retaliation and other uncharacteristic behaviours, which in turn led to punishment by exclusion from school activities [22,24]. One reported doing a friend's homework in return for protection against bullying that he believed was to do with his size [27].

#### Very overweight children are made to feel 'different and terrible'

One clinically obese child who had experienced size-related taunting described feeling, 'fat, you're slow, you're ignorant, you're useless'[[24], p921]. Other responses from overweight children to size-related abuse again emphasised the importance of fitting in socially. One boy said he wore 'baggy T-shirts. I try to hide it' [[22], p233]. Another described how the likelihood of name-calling had made him feel 'different and terrible, like I'm not like everyone else' [[24], p921].

Very overweight children also described difficult experiences with clothes and body-size, including difficulties finding clothes that fit [21], feeling exposed when shopping [24] and having clothes that were the wrong size [22]. They also experienced their bodies as getting in the way of physical activity, either due to physical inability [25,28], name calling when exercising [22], or embarrassment when changing clothes in public [22,24].

### Gender

#### Satisfaction with body size differs between the sexes

Nine studies explored whether girls were more or less satisfied with their body shape than boys [22,26,39-42,45-47]. These found that higher proportions of girls wanted a different body shape from the one they perceived they had, or that more girls than boys described their bodies as 'too heavy' or 'too big'. The girls in these studies consistently wanted their bodies to be 'leaner'. When boys were dissatisfied with their body shape, it was often because they wanted to be 'bulkier'[26,41,42], although the most recent study [39] found 'dissatisfied' boys more often wanted to be thinner, rather than larger.

#### Body size stereotypes vary with gender

As described above, children were critical of generalized representations of overweight people. While two studies found boys to be more negative than girls in all or some of their appraisals of the same figure (e.g., of athletic ability, fitness and eating habits)[36,40], three found no evidence of a difference [26,38,41]. However, responses did differ according to the gender of the body being considered. One study found that bulkier female figures received more negative assessments than leaner ones [38]. In another, children attributed fewer feminine characteristics to overweight female figures [37]. In both studies, the size of male figures made no difference to children's assessments.

#### Checking the robustness of the review: the sensitivity analysis and credibility check with young people

Examination of the contribution of the six lowest quality studies to the two syntheses found that the themes that they contributed to were all supported by a number of other, higher quality studies (for detail see Additional file 7 - online). Therefore, the removal of the lowest quality studies from the synthesis, while it might have changed some of the fine detail, would not have modified the findings to any great extent. Because of the focus of these lower quality studies, the strength of findings relating specifically to girls, very overweight and very young children would be reduced.

The PEAR group of young people who were consulted about the findings of the interpretive synthesis felt that the themes were likely to have covered the most important issues for children. Areas that they felt might be missing were the influence of the media, diversity amongst children and the effectiveness of strategies for achieving and maintaining a healthy size.

Following the consultation, these areas were searched for in the study data, to see if they had been overlooked or had not been given sufficient emphasis. However, children in the studies had not talked about the effectiveness of action aimed at facilitating healthy body sizes, or about diversity amongst children. They had referred in several studies to the size of celebrities and in one study to body sizes in magazines, but these were presented by study authors as though they were only one of many points of discussion. Authors had not described or quoted children as talking specifically about feeling influenced by the media. The data had influenced the development of themes about appropriate and ideal bodies and body comparisons. It did not seem appropriate to turn the idea of media influence into a theme or modify the synthesis structure further.

## Discussion

The UK children whose views were included in this review varied in the extent to which body size was directly relevant to their lives. Children with healthy body sizes did not appear to have this issue high on their everyday agendas. When they were asked directly about body size, being overweight was seen as a problem because of the impact it could have on their lives as social beings, from reduced popularity through to discrimination. The health consequences of obesity appeared to be largely irrelevant. These findings resonate with several recent large-scale consultations that show that children often do not bring up health issues when given a blank sheet on which to identify the priorities for action in their lives [49]. Similarly, it has previously been found that the physical health outcomes of health behaviours are not salient for children, compared with other aspects of their lives, such as friendships and enjoyment [50,51].

Whatever their size, however, these children seemed extremely aware of our society's heightened interest in body size. When asked, many wished their bodies were leaner, sometimes to the extent of aspiring to body weights that would be unattainable for most, as well as unhealthily thin. This dissatisfaction and aspiration for thinness were both seen in other research conducted with children in the UK and elsewhere in the mid-1990s [52,53]. This review's findings suggest that young children, despite often having healthy body sizes, continue to dislike their own bodies.

Negative stereotyping of overweight people has been reported in numerous studies of adults and children in the US, and in UK studies of children aged 11 which were conducted earlier than this review's chronological scope [54]. In several studies in this review, children blamed overweight people for their size and size was represented as something that could be controlled. This emphasis on personal culpability contrasts starkly with widespread understanding in public health of the overriding importance of social and environmental factors largely outside individual control, such as work patterns, transport options and the production and sale of food [1,55]. A reminder that children might have even less control over the factors that affect their body size is provided by a recent review of studies, from the UK and elsewhere, of parents' perceptions about preventing childhood obesity. Amongst the barriers to preventing obesity amongst children identified by parents were, family behaviours, parental attitudes and beliefs, and environmental factors, such as children's schooling and day care arrangements, and parents' access to exercise programmes and other resources [56].

The very overweight children in this review described being teased and bullied on account of their larger size. They reported how this impacted seriously on their well-being and behaviour. A large-scale longitudinal study in the UK found that obese eight-year-old children were one-and-a-half times more likely to have been bullied than average-weight children [57]. Weight-based teasing and bullying has been implicated in weight gain in young people in the US because of its role in increased unhealthy eating and reduced physical activity [58]. It is notable that the children in this review commented on the negative impact of size-related abuse in relation to getting changed for, or taking part in physical activity. It is likely that very overweight children in the UK who attempt to exercise as a way of reducing their body size will experience a major barrier in the shape of humiliation and size-related teasing from some of their peers.

The aspirations of girls and boys in this review for their bodies differed, and girls were consistently more dissatisfied with their size. The interest in a 'lean' body shape seen among girls in this review resonates with findings from earlier research in the UK and elsewhere [53], and is in line with 'feminine' ideals presented in the media [59,60]. Boys' interest in fitness and muscles and the aspiration of some for bulkier body shapes suggests the influence of ideals around male muscularity [61]. While girls' aspirations are still more likely to raise concern, a large proportion of boys also aspired to very thin body shapes in the most recent study found during this review. This might be an indicator that unhealthily thin body size ideals should no longer be seen as limited to girls and young women.

### Strengths and limitations of this review

This is the first review of which we are aware that seeks out, appraises and synthesizes in a systematic way the findings from studies of children's views about body size. As with all reviews, it is possible that this one has missed some relevant literature, and it is impossible to gauge the impact that this might have had on its findings. However, to reduce the likelihood that we have missed studies, very sensitive searches of bibliographic databases were supplemented by other methods to seek out literature that can be hard to find, such as theses and unpublished reports. Since sources often cover literature from the US better than they do other countries, specific searches of UK-rich data sources were also conducted so as to increase our yield of UK literature.

The review is, however, limited by the methodological quality of existing research. Findings about children's views about body size were relatively scarce and were not presented in much depth or with much contextual detail. Few of the studies described taking many steps to ensure rigour and increase confidence in the quality of their findings. Adding some face validity to our findings is the fact that our consultation did not identify any themes that appeared unexpected to a group that, relatively recently, would have been the same age as children studied in the review (the PEAR group). The findings of the aggregative synthesis also add confidence about the generalizability of some of the findings in the interpretive review, in particular the disparity between boys' and girls' satisfaction with their body sizes. However, many of the studies in this synthesis were small and limited in their reporting of how their samples were constructed. While it is clear that a range of children had been involved in many of the studies, some appear to be underrepresented, in particular young children and those who were socio-economically disadvantaged or not at school.

Furthermore, very few studies in this review reported using approaches that privilege children's own framing of issues in their lives or started from the position that children themselves may usefully contribute ideas and analyses to help develop theories about their own lives and the questions asked of them. The studies often aimed primarily to explore or test existing theories. Frameworks used to analyse children's responses also often appeared to have been developed without any consultation or collaboration with children themselves.

As a result, many of the views expressed and reported in the studies in this review may be constrained by adult preconceptions as to what might be important to children. It is unclear what kinds of insights or emphases the children in these studies might have offered if they had been fully enabled to present or consider their own perspectives. It is also possible that the children in some of the studies may have been saying what they thought adult researchers or other children present with them in focus groups wanted to hear. Some children made it clear that they were aware of contradictions between the seemingly benign and neutral statements that children and others make about large body size, and the less-than-neutral actions that are then taken; for example, people saying that size does not matter, but then discriminating on this basis. The highly social nature of body size, coupled with evidence of discomfort and anxiety amongst some children around this topic, point to the need to take extreme care when constructing research environments for children to discuss this issue.

The facilitated exchange of ideas between two children in the Cole and colleagues' study (described above under 'A large body size means you are...') illustrates how children's views can be nuanced [33]. This study's data collection methods built upon existing relationships between the children themselves, and between the children and the research team. The methods included observational, drawing and play techniques and provide an example of one way in which researchers might help children describe and analyse their own lives. Such studies are likely to be small-scale, because of the need for careful attention to the relationships central to data collection [9]. There is an urgent need to conduct qualitative research that combines the use of these kinds of child-friendly, or co-constructive, data collection methods, with rigorous data analysis and thorough reporting of all methods, in order to strengthen the evidence-base.

## Conclusions

This systematic review has identified a disparate set of qualitative and quantitative studies containing data from UK children about their views and experiences of body size, shape and weight. It has synthesized this into a coherent whole that explores children's views in the areas of: how body size matters; desirable and acceptable bodies; embodied experiences of body size; and gender. However, in only a few of the studies had attempts been made to encourage children's own analyses, or sufficient efforts been taken to encourage children's full engagement and participation in research on this topic. The studies did not fully represent children's diversity, and so seriously restrict any analysis of variations in perspective between children. A strong evidence-base for policy on children and body size would include findings from good quality research about children's views, since children have direct experience and a considerable stake in the matter. This review indicates however, that research with children on their views on this topic needs to be far more rigorous and equitable than is currently the case.

## Competing interests

The authors declare that they have no competing interests.

## Authors' contributions

The review protocol was developed by RR, JT and JW. RR and JW conducted searches and screened studies. RR, JW and KO developed the data extraction tool. Data extraction, quality appraisal and synthesis were conducted by RR and KO. KO conducted the consultation with the NCB PEAR group. All authors contributed to the writing of this paper.

## Pre-publication history

The pre-publication history for this paper can be accessed here:

http://www.biomedcentral.com/1471-2458/11/188/prepub
